# Different Master Regulators Define Proximal and Distal Gastric Cancer: Insights into Prognosis and Opportunities for Targeted Therapy

**DOI:** 10.3390/curroncol32080424

**Published:** 2025-07-28

**Authors:** Luigi Marano, Salvatore Sorrenti, Silvia Malerba, Jaroslaw Skokowski, Karol Polom, Sergii Girnyi, Tomasz Cwalinski, Francesco Paolo Prete, Alejandro González-Ojeda, Clotilde Fuentes-Orozco, Aman Goyal, Rajan Vaithianathan, Miljana Vladimirov, Eleonora Lori, Daniele Pironi, Adel Abou-Mrad, Mario Testini, Rodolfo J. Oviedo, Yogesh Vashist

**Affiliations:** 1 Department of Medicine, Academy of Applied Medical and Social Sciences-AMiSNS (Akademia Medycznych I Spolecznych Nauk Stosowanych), 52-300 Elbląg, Poland; jskokowski@copernicus.gda.pl (J.S.); k.polom@amisns.edu.pl (K.P.); y.vashist@kfu.edu.sa (Y.V.); 2 Department of General Surgery and Surgical Oncology, “Saint Wojciech” Hospital, “Nicolaus Copernicus” Health Center, 80-000 Gdańsk, Poland; sgirnyi@copernicus.gda.pl (S.G.); tcwalinski@copernicus.gda.pl (T.C.); 3 Department of Surgery, Dnipro State Medical University, 49044 Dnipro, Ukraine; 4 Department of Medicine, Surgery, and Neurosciences, University of Siena, 53100 Siena, Italy; 5 Department of Surgery, “Sapienza” University of Rome, 00161 Roma, Italy; salvatore.sorrenti@uniroma1.it; 6 Department of Precision and Regenerative Medicine and Ionian Area, University of Bari “Aldo Moro”, 70110 Bari, Italy; s.malerba10@studenti.uniba.it (S.M.); francesco.prete@uniba.it (F.P.P.); mario.testini@uniba.it (M.T.); 7 Department of Surgery, Centro Médico Nacional de Occidente IMSS, Guadalajara 44329, Mexico; agonzalez261@ucol.mx (A.G.-O.); clotilde.fuentes@imss.gob.mx (C.F.-O.); 8 Department of General Surgery, Mahatma Gandhi Medical College and Research Institute, Puducherry 607402, India; doc.aman.goyal@gmail.com (A.G.); rajan.vaithianathan@sbvu.ac.in (R.V.); 9 Department of Medicine, Adesh Institute of Medical Sciences and Research, Bathinda 151001, India; 10 Department of Surgery, University Bielefeld—Campus Lippe, 32756 Detmold, Germany; miljana.vladimirov@klinikum-lippe.de; 11 Department of Surgery, Sapienza University of Rome, AOU Policlinico Umberto I, 00161 Rome, Italy; eleonora.lori@uniroma1.it (E.L.); daniele.pironi@uniroma1.it (D.P.); 12 Department of Surgery, Centre Hospitalier Universitaire d’Orléans, 45000 Orléans, France; adel.abou-mrad@orange.fr; 13 Department of Surgery, Nacogdoches Medical Center, Nacogdoches, TX 75962, USA; roviedo3@central.uh.edu; 14 Department of Surgery, Tilman J. Fertitta Family College of Medicine, University of Houston, Houston, TX 77001, USA; 15 Department of Surgery, Sam Houston State University College of Osteopathic Medicine, Conroe, TX 77301, USA; 16 Department of Surgical Oncology, Asklepios Harz Clinic Goslar, 38640 Goslar, Germany

**Keywords:** gastric cancer, master regulators, FOXM1, CDX2, tumor localization, transcriptomics, survival analysis

## Abstract

*Background:* Gastric cancer (GC) represents a significant global health burden with considerable heterogeneity in clinical and molecular behavior. The anatomical site of tumor origin—proximal versus distal—has emerged as a determinant of prognosis and response to therapy. The aim of this paper is to elucidate the transcriptional and regulatory differences between proximal gastric cancer (PGC) and distal gastric cancer (DGC) through master regulator (MR) analysis. *Methods:* We analyzed RNA-seq data from TCGA-STAD and microarray data from GEO (GSE62254, GSE15459). Differential gene expression and MR analyses were performed using DESeq2, limma, corto, and RegEnrich pipelines. A harmonized matrix of 4785 genes was used for MR inference following normalization and batch correction. Functional enrichment and survival analyses were conducted to explore prognostic associations. *Results:* Among 364 TCGA and 492 GEO patients, PGC was associated with more aggressive clinicopathological features and poorer outcomes. We identified 998 DEGs distinguishing PGC and DGC. PGC showed increased FOXM1 (a key regulator of cell proliferation), STAT3, and NF-κB1 activity, while DGC displayed enriched GATA6, CDX2 (a marker of intestinal differentiation), and HNF4A signaling. Functional enrichment highlighted proliferative and inflammatory programs in PGC, and differentiation and metabolic pathways in DGC. MR activity stratified survival outcomes, reinforcing prognostic relevance. *Conclusions:* PGC and DGC are governed by distinct transcriptional regulators and signaling networks. Our findings provide a biological rationale for location-based stratification and inform targeted therapy development.

## Simple Summary

This study shows proximal and distal gastric cancers are biologically distinct. Proximal tumors activate FOXM1 and STAT3, linked to poor survival, while distal tumors express CDX2 and GATA6. These regulators influence prognosis and suggest targets for personalized treatment.

## 1. Introduction

Gastric cancer (GC) continues to be a significant global health concern. In 2022, approximately 968,784 new cases were reported worldwide, positioning GC as the fifth most common malignancy, with male predominance [1]. Despite advancements in diagnostic and therapeutic strategies, the overall five-year relative survival rate for GC remains around 36% [2]. Traditional classification systems—such as Lauren’s and the WHO histological subtypes—categorize GC into intestinal, diffuse, and mixed types [3,4], but these fail to capture the significant intratumoral and intertumoral heterogeneity that characterizes GC. Increasing evidence suggests that the anatomical location within the stomach—proximal (upper third, including the esophagogastric junction and gastric cardia) versus distal (lower third, including the antrum and pylorus)—plays a pivotal role in determining the tumor’s clinicopathological features, biological behavior, and response to therapy [5].

Several studies highlight key differences between proximal and distal gastric cancers in epidemiology, histopathology, and prognosis. Proximal tumors often show larger size, more submucosal invasion, and undifferentiated histology, with some evidence suggesting worse survival outcomes compared to distal gastric cancers [6,7,8].

Recent genomic and transcriptomic studies have begun to reveal the molecular basis of this heterogeneity, suggesting that different transcriptional programs govern tumorigenesis in proximal versus distal gastric cancer. Master regulators (MRs)—transcription factors or regulatory genes that orchestrate the activity of large gene networks—are emerging as critical determinants of cancer phenotypes [9,10,11].

Investigating the differential activity of MRs in proximal versus distal GCs could provide valuable insights into the distinct biological behaviors observed between these tumor locations. Previous studies comparing proximal and distal GC subtypes have predominantly focused on histopathologic or mutational differences, often without reconstructing transcriptional regulatory networks or assessing master regulator (MR) activity in a systematic manner [12,13,14]. This lack of network-level integration limits our understanding of the transcriptional logic and biological programs driving location-specific tumor behavior. Therefore, this study aims to identify differentially expressed genes (DEGs) associated with tumor localization, reconstruct transcriptional regulatory networks to identify location-specific MRs, and correlate MR activity with clinicopathological parameters and patient survival, contributing to more personalized and effective therapeutic strategies.

## 2. Methods

### 2.1. Data Acquisition

For this investigation into the association between tumor localization (proximal vs. distal) and MR activity in GC, we utilized publicly accessible datasets from The Cancer Genome Atlas (TCGA) and the Gene Expression Omnibus (GEO).

- **The Cancer Genome Atlas (TCGA):** We accessed RNA sequencing (RNA-seq) data and corresponding clinical annotations for GC patients from the Genomic Data Commons (GDC) Data Portal (https://portal.gdc.cancer.gov/ accessed on 15 February 2025). Specifically, the TCGA Stomach Adenocarcinoma (TCGA-STAD) project was utilized. The GDC provides harmonized genomic data, facilitating integrative analyses across various studies. Tumor localization in TCGA was assigned based on clinical metadata fields, including “primary site” and “site of resection or biopsy.” Tumors located at the gastroesophageal junction or cardia were classified as proximal gastric cancer, while those involving the antrum or pylorus were categorized as distal gastric cancer.
- **Gene Expression Omnibus (GEO):** We retrieved microarray-based gene expression datasets GSE62254 and GSE15459 (https://www.ncbi.nlm.nih.gov/geo/ accessed on 1 March 2025), which include comprehensive clinical annotations for GC patients.

### 2.2. Patient Cohort Selection

- Inclusion criteria for this study were as follows:
- Histologically confirmed diagnosis of gastric adenocarcinoma.
- Availability of tumor localization data, categorizing tumors as proximal (esophagogastric junction and cardia) or distal (antrum and pylorus).
- Comprehensive clinical and gene expression data.

From the TCGA-STAD cohort, we identified patients meeting these criteria. Similarly, patients from the GSE62254 and GSE15459 datasets with specified tumor localization were included. This stratification enabled a comparative analysis of molecular profiles between proximal and distal GC cases.

### 2.3. Data Preprocessing and Normalization

For TCGA RNA-seq data, raw counts were normalized using the Transcripts Per Million (TPM) method to account for sequencing depth and gene length. Microarray data from GEO datasets were preprocessed and normalized using the Robust Multi-array Average (RMA) algorithm. To mitigate potential batch effects arising from combining data across different platforms and studies, we employed the ComBat function from the Surrogate Variable Analysis (sva) package in R (version 4.3.2; R Foundation for Statistical Computing, Vienna, Austria) [15]. Prior to normalization, we conducted standard quality control procedures across all datasets. Principal component analysis was employed to detect and exclude expression outliers, defined as samples falling beyond 3 standard deviations from the centroid of the first two principal components. Additionally, samples with incomplete clinical annotations or missing expression data for more than 25% of genes were excluded from downstream analyses.

### 2.4. Differential Expression Analysis

We conducted differential gene expression analysis between proximal and distal GC groups using the limma package in R (version 4.3.2; R Foundation for Statistical Computing, Vienna, Austria) [16]. Genes exhibiting an adjusted *p*-value < 0.05 and absolute log2 fold change > 1.5 were considered significantly differentially expressed.

### 2.5. Master Regulator Inference

To identify MRs—transcription factors or genes that orchestrate gene expression programs associated with tumor localization—we utilized the corto package in R (version 4.3.2; R Foundation for Statistical Computing, Vienna, Austria) [17]. We selected corto and RegEnrich due to their complementary methodological frameworks: corto reconstructs co-expression networks using mutual information, while RegEnrich integrates differential expression with network topology and regulatory influence to identify key transcriptional regulators. Both tools are computationally efficient, and widely adopted in transcriptomic studies. This tool constructs gene regulatory networks and identifies key regulators based on mutual information and enrichment analyses. MRs were ranked according to their regulon activity scores, highlighting those with significant differential activity between proximal and distal tumors.

### 2.6. Functional Enrichment Analysis

We performed Gene Ontology (GO) and Kyoto Encyclopedia of Genes and Genomes (KEGG) pathway enrichment analyses on the target genes regulated by identified MRs using the clusterProfiler package in R (version 4.3.2; R Foundation for Statistical Computing, Vienna, Austria) [18]. This approach elucidated the biological processes and pathways predominantly influenced by MRs in the context of tumor localization.

### 2.7. Statistical Analysis

To assess the relationship between tumor localization (proximal vs. distal) and various clinical variables, we employed the chi-square test for independence. This analysis was conducted using Jamovi (version 2.6.44; The jamovi project, Sydney, Australia). Significant associations were visualized using Jamovi’s built-in plotting capabilities.

For survival analyses, we utilized the Cox proportional hazards model. The survival analyses were performed using JASP (version 0.18.3; JASP Team, Amsterdam, The Netherlands). A stepwise procedure was applied to identify the most significant variables for the final Cox models, and the results were presented as forest plots generated within JASP. Clinical covariates included in the multivariate models were selected based on their biological relevance and statistical significance (*p* < 0.10) in univariate Cox analyses. This ensured that only clinically meaningful and prognostically informative variables were retained.

Differential gene expression analysis between proximal and distal gastric cancer samples was performed using the DESeq2 package in R (version 4.3.2; R Foundation for Statistical Computing, Vienna, Austria) [19]. Genes with an adjusted *p*-value < 0.05 and an absolute log2 fold change > 0.58 (corresponding to an absolute fold change greater than 1.5) were considered significantly differentially expressed.

To identify biological pathways and processes associated with DEGs, we conducted gene set enrichment analyses using the ClusterProfiler package in R (version 4.3.2; R Foundation for Statistical Computing, Vienna, Austria). This analysis encompassed the Molecular Signatures Database (MSigDB) Hallmark gene sets [20], Gene Ontology (GO) terms, Kyoto Encyclopedia of Genes and Genomes (KEGG) pathways, and other relevant gene sets [21,22]. Results with a false discovery rate (FDR) adjusted *p*-value < 0.05 were considered statistically significant.

For master regulator analysis (MRA), we utilized the RegEnrich package in R (version 4.3.2; R Foundation for Statistical Computing, Vienna, Austria), which integrates gene expression data with regulatory network information to identify key transcriptional regulators driving observed gene expression changes. RegEnrich calculates enrichment scores for each regulator, reflecting their influence on the gene expression profile. Significant master regulators were identified based on their enrichment scores and associated *p*-values.

Furthermore, univariate Cox proportional hazards analyses were conducted to evaluate the impact of MR activity on OS. The normalized enrichment scores (NES) of MRs were used as continuous variables in these models. Results were summarized in forest plots to illustrate the hazard ratios and confidence intervals for each MR.

## 3. Results

### 3.1. Patient Characteristics

A total of 364 patients from the TCGA-STAD cohort and 492 patients from the GSE62254 (n = 300) and GSE15459 (n = 192) datasets were included. Among them, 207 were classified as having PGC and 649 as DGC. Comparative analysis of clinical parameters revealed a higher proportion of poorly differentiated tumors, signet-ring cell histology, and deeper invasion (T3/T4) among PGC cases. DGC cases were more often of the intestinal subtype and exhibited better differentiation grades (*p* < 0.01). Lymph node involvement (N-stage) and distant metastasis rates were also significantly different between groups (*p* < 0.05), with proximal tumors showing a more aggressive pattern. Table 1 summarizes the key clinical differences between PGC and DGC groups.

**Table 1:** Clinical characteristics of proximal vs. distal gastric cancers.

Characteristic	Proximal GC (n = 207)	Distal GC (n = 649)	*p*-Value	OR (95% CI)
Diffuse histology	135 (65%)	227 (35%)	<0.001	3.59 (2.64–4.89)
Poorly differentiated	149 (72%)	311 (48%)	<0.001	2.86 (2.06–3.98)
T3/T4 stage	141 (68%)	318 (49%)	<0.001	2.26 (1.64–3.12)
N+ lymph node involvement	124 (60%)	273 (42%)	0.014	2.06 (1.50–2.83)
Metastasis (M1)	37 (18%)	65 (10%)	0.021	1.98 (1.27–3.08)
Intestinal subtype	50 (24%)	493 (76%)	<0.001	0.14 (0.10–0.20)

Comparison of clinicopathological features between proximal gastric cancer (PGC) and distal gastric cancer (DGC) cohorts. PGC cases exhibited significantly higher frequencies of diffuse histology, poor differentiation, deeper tumor invasion (T3/T4), and lymph node involvement. Conversely, DGC was predominantly intestinal subtype with more favorable differentiation. *p*-values were calculated using Chi-square or Fisher’s exact test, as appropriate.

### 3.2. Differential Gene Expression Analysis

Using DESeq2 and limma, we identified 998 differentially DEGs between PGC and DGC samples. DESeq2 was applied to the RNA-seq data from TCGA, while limma was used for the microarray data from GEO (GSE62254 and GSE15459). The analyses were performed separately for each dataset and the significant DEGs were subsequently integrated for comparative interpretation (adjusted *p*-value < 0.05, |log2FC| > 1.5). This integrative strategy ensured that the reported 998 DEGs represent cross-validated genes consistently distinguishing PGC from DGC, minimizing platform-specific artifacts. Among these, 486 genes were significantly upregulated in PGC, including genes related to EMT (ZEB1, SNAI2), inflammation (IL1B, TNFAIP3), immune modulation (PD-L1, CXCL9), and stemness (PROM1, LGR5). In contrast, 512 genes were upregulated in DGC, notably those involved in epithelial differentiation (TFF1, GKN1), mucin production (MUC5AC), metabolic activity (G6PC), and tight junctions (CLDN18). Differential gene expression analysis between proximal and distal gastric cancer samples was conducted using dataset-specific tools: DESeq2 for RNA-seq data from the TCGA cohort, and limma for microarray data from the GEO datasets (GSE62254 and GSE15459). DESeq2 is a widely used tool for analyzing count-based NGS data, providing methods to test for differential expression by modeling the data with a negative binomial distribution. Genes with an adjusted *p*-value < 0.05 and an absolute log2 fold change > 0.58 (corresponding to an absolute fold change greater than 1.5) were considered significantly differentially expressed. From the TCGA-STAD cohort, we identified patients meeting these criteria. Similarly, patients from the GSE62254 and GSE15459 datasets with specified tumor localization were included. This stratification enabled a comparative analysis of molecular profiles between proximal and distal GC cases.

### 3.3. Identification of Location-Specific Master Regulators

We applied both corto and RegEnrich pipelines to infer regulatory networks and identify MRs. corto identified 112 candidate MRs, of which 21 exhibited significant differential activity (*p* < 0.01) between PGC and DGC. Among the 21 significantly differentially active MRs identified (*p* < 0.01), representative normalized enrichment scores (NES) were as follows: in proximal tumors, FOXM1 (NES = 2.43, *p* = 3.1 × 10^−4^), STAT3 (NES = 2.21, *p* = 6.7 × 10^−4^), and NF-κB1 (NES = 2.15, *p* = 8.2 × 10^−4^) exhibited strong positive enrichment, consistent with pro-tumorigenic and inflammatory activity. In contrast, distal tumors showed significantly negative enrichment for CDX2 (NES = −2.44, *p* = 3.5 × 10^−4^), GATA6 (NES = −2.36, *p* = 4.8 × 10^−4^), and HNF4A (NES = −2.18, *p* = 6.1 × 10^−4^), supporting their roles in epithelial differentiation and mucosal identity.

### 3.4. Functional Enrichment of MR Target Genes

We conducted GO and KEGG enrichment analysis on MR target regulons. In PGC, enriched pathways included cell cycle progression (GO:0007049), cytokine-mediated signaling (GO:0019221), NF-κB signaling (KEGG:04064), and ECM remodeling (KEGG:04512). DGC-associated MRs were linked to metabolic regulation (GO:0008152), epithelial morphogenesis (GO:0002009), mucosal defense, and gastric acid secretion (KEGG:04971).

Notably, proximal-specific FOXM1 targets were heavily enriched in mitotic spindle and G2/M checkpoint modules, suggesting a proliferative tumor phenotype. CDX2 regulons in distal tumors were significantly enriched for transcriptional networks regulating intestinal metaplasia and secretory lineage maintenance.

### 3.5. Correlation of MR Activity with Clinical and Survival Outcomes

We performed univariate and multivariate Cox proportional hazards analyses using normalized enrichment scores (NES) of MRs. In the TCGA cohort, elevated activity of FOXM1 (HR = 1.82, 95% CI: 1.31–2.52, *p* = 0.001) and STAT3 (HR = 1.56, 95% CI: 1.12–2.17, *p* = 0.007) were independently associated with reduced overall survival (OS). Similarly, NF-κB1 activity was significantly linked to lymph node metastasis (*p* = 0.003).

**Figure 1:**
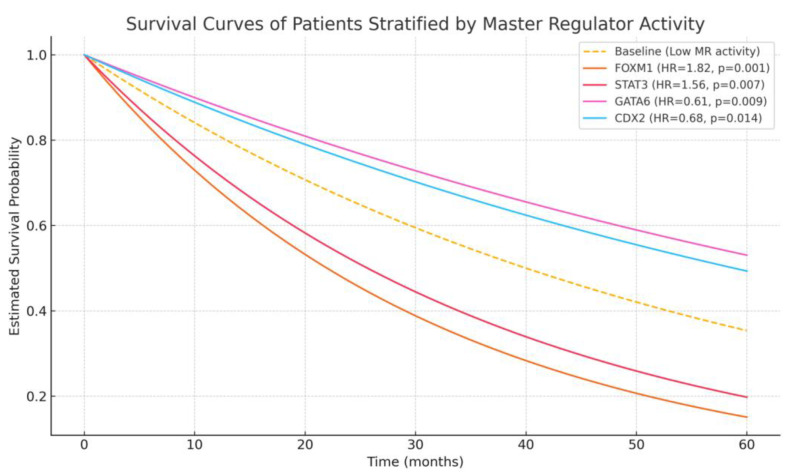
Survival curves of patients stratified by master regulator activity. Kaplan–Meier survival curves illustrating overall survival probabilities over time for patients stratified by master regulator (MR) activity levels. Elevated FOXM1 (HR = 1.82, *p* = 0.001) and STAT3 (HR = 1.56, *p* = 0.007) activities were associated with reduced survival, while higher GATA6 (HR = 0.61, *p* = 0.009) and CDX2 (HR = 0.68, *p* = 0.014) activities predicted improved outcomes. Baseline curve represents low MR activity.

**Figure 2:**
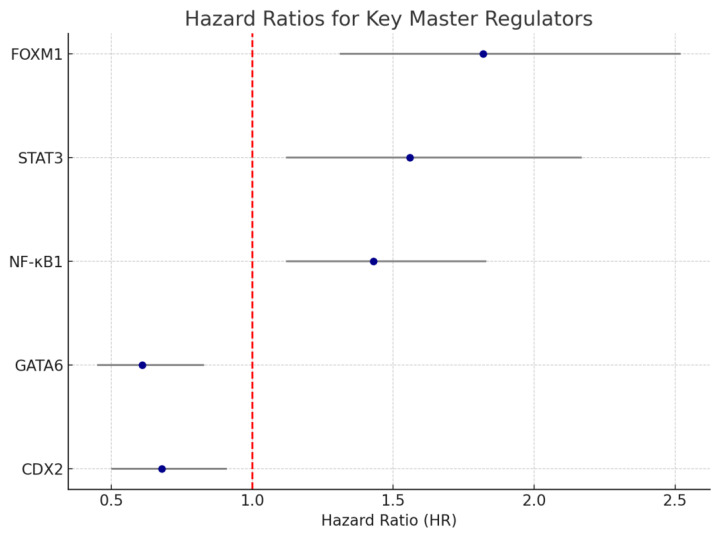
Forest plot of hazard ratios for key master regulators. Summary of forest plot illustrating the hazard ratios (HRs) and 95% confidence intervals (CIs) for master regulators significantly associated with overall survival in the TCGA cohort. FOXM1, STAT3, and NF-κB1 were associated with poor prognosis (HR > 1), while GATA6 and CDX2 were linked to favorable outcomes (HR < 1). These findings were adjusted for confounding clinical variables in multivariate Cox regression models. The red dashed line indicates the null value (HR = 1), where no effect is observed.

In contrast, high GATA6 (HR = 0.61, *p* = 0.009) and CDX2 (HR = 0.68, *p* = 0.014) activity were associated with favorable OS, particularly in DGC patients (Figure 1). These findings remained significant after adjusting for confounders, including stage, histology, and treatment modality. A forest plot summarizing hazard ratio and 95% confidence intervals for key MRs is provided in Figure 2.

## 4. Discussion

This study reveals that proximal and distal gastric cancers exhibit distinct molecular and clinical profiles. Notably, using two complementary MR analysis pipelines (corto and RegEnrich), we uncovered location-specific key transcriptional regulators. PGC tumors showed upregulation of oncogenic MRs such as FOXM1 and STAT3, while DGC tumors were enriched for MRs involved in differentiated gastric/intestinal lineage, including CDX2 and GATA6. These findings were mirrored by pathway enrichment analyses: PGC was characterized by activation of NF-κB signaling and cell cycle/proliferation pathways, whereas DGC exhibited enrichment for metabolic processes and epithelial morphogenesis pathways, consistent with prior reports linking distal tumors to enhanced oxidative metabolism and differentiation-associated metabolic reprogramming [23,24,25]. Clinically, the location-specific MR activity had prognostic correlates—high FOXM1 and STAT3 expression (predominant in PGC)—were associated with poorer overall and disease-specific survival, whereas elevated GATA6 and CDX2 (predominant in DGC) correlated with more favorable patient outcomes. Proximal and distal gastric cancers are biologically distinct subtypes, driven by different regulatory networks and clinical behaviors, aligning with known differences in their epidemiologic and genetic features based on tumor location [26,27]. For example, proximal GC has been rising in incidence in Western countries and is linked to obesity and gastroesophageal reflux, whereas distal GC remains more associated with Helicobacter pylori infection and intestinal metaplasia [14]. Our transcriptional findings provide a molecular rationale for these epidemiologic differences and align with prior evidence that tumor location is an important prognostic factor in gastric cancer [27,28,29]. In a meta-analysis of 50 studies, Petrelli et al. [27] reported that cancers of the gastric cardia/GEJ carry ~30–40% higher mortality risk than distal tumors, leading to the recommendation that proximal tumors should be recognized as a distinct, high-risk group in need of tailored strategies. Our results build upon this by implicating specific transcriptional drivers (FOXM1, STAT3 vs. GATA6, CDX2) that may underlie the more aggressive clinical course of PGC relative to DGC.

The identification of FOXM1 and STAT3 as top MRs in proximal gastric tumors highlights a more proliferative and inflammatory oncogenic program in PGC [30]. FOXM1 is a well-known cell cycle regulator frequently upregulated in cancers; our finding that FOXM1 is elevated in PGC is in line with its association with advanced disease and poor outcomes in gastric cancer [31,32]. FOXM1 overexpression has been shown to drive tumor growth and chemoresistance in GC, making it an attractive therapeutic target in aggressive gastric tumors [31]. Similarly, STAT3 is a transcription factor activated by cytokine signaling (e.g., IL-6) and chronic inflammation [33,34,35,36]. Higher STAT3 activity in proximal tumors links to inflammation-driven tumor microenvironment, advanced stage, and poorer survival [37]. Notably, STAT3 can upregulate EZH2 and other oncogenic effectors in GC, promoting proliferation and immune evasion [37]. The enrichment of NF-κB signaling in PGC further supports the notion of a pro-inflammatory milieu in these tumors. NF-κB is a master mediator of inflammation-induced tumorigenesis, and its activation in PGC may stem from distinct etiologic factors (for instance, reflux-related esophagitis or obesity-associated cytokines) that are more prominent in cardia tumors [14,38]. Although inflammation is a shared feature across gastric cancer subtypes, the origin and nature of inflammatory stimuli differ between anatomical sites. In DGC, *Helicobacter pylori* infection plays a central etiological role, initiating chronic gastritis and promoting an inflammatory milieu characterized by cytokines such as IL-1β, TNF-α, and IL-8, which contribute to mucosal damage and carcinogenesis. In contrast, PGC is more frequently associated with reflux-mediated injury, where bile acids and gastric acid exposure activate NF-κB and STAT3 signaling pathways, leading to the upregulation of IL-6, IL-8, and other pro-inflammatory mediators. These site-specific inflammatory contexts may underlie the preferential activation of inflammatory master regulators such as STAT3 and NF-κB1 in PGC observed in our study, reflecting a cytokine landscape shaped more by chemical reflux than microbial infection [14,38]. These data suggest that proximal tumors may benefit from therapies targeting inflammatory and cell cycle pathways.

For example, JAK/STAT3 inhibitors are under development and have shown early promise in gastric cancer patients [27,39,40,41,42,43], and FOXM1 inhibitors or cell cycle modulators could potentially be explored to specifically curb the proliferative drive in PGC [43,44,45]. The clinical relevance of these MRs is underscored by our survival analyses: patients with PGC-like MR profiles (high FOXM1/STAT3) had significantly worse overall survival and disease-specific survival, reinforcing that these factors could serve as prognostic biomarkers or therapeutic targets. Indeed, our findings provide a biological explanation for the poorer outcomes of proximal gastric cancer patients reported in clinical series [27].

In contrast, DGC appears to be governed by a different transcriptional program more related to gastric-intestinal differentiation and metabolic homeostasis. CDX2, a caudal-type homeobox gene, was identified as a key regulator in DGC [23,46,47]. CDX2 is normally a marker of intestinal epithelial identity; its expression in gastric tumors is generally associated with the intestinal histologic subtype and the presence of intestinal metaplasia in surrounding mucosa [48]. Consistent with our results, CDX2-positive gastric cancers have been reported to be less aggressive, showing better differentiation, lower invasive stage, and markedly improved survival compared to CDX2-negative cancers [49]. A meta-analysis of over 1500 patients found that CDX2-positive tumors had a significantly higher 5-year survival rate (HR ~0.45 for mortality) and tended to be of lower stage and grade [49]. In our DGC cohort, high CDX2 expression likewise correlated with favorable prognosis, suggesting that distal tumors often retain features of intestinal differentiation that confer a less malignant phenotype. Another prominent MR in DGC was GATA6, a transcription factor involved in endodermal lineage development. GATA6’s role in GC appears context-dependent. Some studies have identified GATA6 as an oncogenic driver—for instance, GATA6 amplifications can promote tumor growth and metastasis in upper gastrointestinal cancers [50,51,52]. On the other hand, our finding of increased GATA6 in DGC (the subset with better outcomes) aligns with evidence that loss of GATA6 activity is associated with tumor progression. Wu et al. reported that GATA6 is frequently silenced by promoter hypermethylation in gastric cancer, and patients with higher GATA6 methylation (indicative of low GATA6 expression) have significantly shorter survival [53]. Thus, GATA6 may function as a differentiation factor in certain gastric tumors, and its presence in distal tumors could help maintain an epithelial phenotype that restrains aggressiveness [54,55]. It is noteworthy that GATA6 and CDX2 were both enriched in our DGC analysis—this combination likely reflects the intestinal-type gastric cancers that commonly arise in the distal stomach. According to the Lauren classification [3] and WHO definitions [4], intestinal-type gastric tumors are typically well differentiated and develop in the context of chronic inflammation and intestinal metaplasia. This histological subtype is associated with a distinct molecular profile characterized by elevated expression of *CDX2* and *GATA6*, and, consistent with our findings, shows enrichment in morphogenetic and metabolic pathways. Biologically, distal tumors may rely more on metabolic adaptations to the atrophic gastric environment (for example, altered acid secretion and microbiota in the distal stomach) and on maintaining glandular architecture, whereas proximal tumors prioritize proliferation and inflammation-driven invasion. These distinct regulatory programs suggest that gastric cancers should not be treated as a uniform entity; rather, location-specific biomarkers like CDX2 or GATA6 could potentially inform prognosis and therapeutic decisions. For instance, CDX2 expression might identify patients likely to have a more indolent disease course [7], while absence of such differentiation markers in a tumor might prompt more aggressive management.

Tumor location influences prognosis and treatment; PGC and DGC differ biologically, activating distinct master regulators with therapeutic potential drug targets [25]. The elevated FOXM1 and STAT3 activity in PGC raises the possibility of using agents that inhibit these pathways in proximal tumors—examples include STAT3 pathway inhibitors (some of which have entered early-phase clinical trials) [56] or indirect suppression of FOXM1 through cell cycle kinase inhibitors [57,58,59]. Conversely, the presence of CDX2 and GATA6 in DGC suggests that distal tumors retain a degree of differentiation; these tumors might respond better to therapies that exploit their slower growth or that induce differentiation. This study draws a parallel between gastric cancer and colorectal cancer “sidedness,” suggesting tumor location may influence therapy response and outcomes.

For example, it would be worthwhile to retrospectively stratify clinical trial data by proximal vs. distal tumor and see if targeted therapies (like anti-HER2, immune checkpoint inhibitors, or anti-angiogenic agents) show differential efficacy. Additionally, recognizing the prominent NF-κB and STAT3 signaling in PGC, one might hypothesize that these tumors could have a more immunosuppressive or cytokine-rich microenvironment, which could influence response to immunotherapy. This transcriptional dichotomy may hold implications for immunotherapeutic stratification. Proximal tumors, enriched in STAT3 and NF-κB-driven inflammation and immune evasion programs, may represent candidates for combinatorial immunotherapy targeting inflammatory checkpoints. For instance, retrospective analyses of KEYNOTE-059 [60] and ATTRACTION-2 [61] trials have suggested enhanced responsiveness to PD-1 blockade in tumors with inflamed, IFN-γ-rich microenvironments, which are features associated with STAT3 activity. Conversely, distal tumors with dominant GATA6 and CDX2 programs may reflect a more epithelial, immune-excluded phenotype less amenable to immune checkpoint inhibition. Thus, integrating MR activity with anatomical classification could inform patient selection in future trials exploring location-tailored immunotherapeutic strategies. While we did not perform formal immune cell deconvolution using tools such as CIBERSORT or xCell, the activation of STAT3 and NF-κB signaling in PGC provides strong transcriptional evidence for an inflammation-associated tumor microenvironment. Both STAT3 and NF-κB are canonical mediators of tumor-promoting inflammation, and their elevated activity in proximal tumors supports the presence of a cytokine-rich, immune-active milieu. Furthermore, key inflammatory mediators such as IL1B and TNFAIP3 were overexpressed in PGC, and gene ontology analyses showed enrichment for cytokine signaling pathways. Although cell-type-level resolution was beyond the scope of the present study, our findings strongly suggest that PGCs are embedded in a more pro-inflammatory TME. We acknowledge that future studies incorporating immune deconvolution or direct immunophenotyping will be essential to validate and extend these observations. An important methodological consideration is the integration of data from different platforms (TCGA RNA-seq vs. GEO microarray). We addressed potential batch effects by normalizing each dataset separately (TPM for RNA-seq, RMA for microarray) and then applying the ComBat algorithm (from the sva package) to adjust for platform-related differences. Differential expression analyses were conducted within each cohort (using DESeq2 for TCGA and limma for GEO) and combined at the interpretation stage rather than by merging raw data. This strategy mitigates many technical biases in cross-dataset comparisons, although we acknowledge that subtle platform differences could persist. Notably, the key DEGs and master regulators emerged consistently from both the TCGA and GEO analyses, suggesting that our biological conclusions are robust despite the use of heterogeneous data sources.

While this analysis provides novel insights, several limitations should be acknowledged. First, our study is retrospective in nature, predominantly leveraging public gene expression datasets (e.g., from TCGA and GEO) and corresponding clinical annotations. As a result, inherent biases in patient selection or differences in data generation (batch effects) could influence the results. The definition of “proximal” vs. “distal” was based on dataset annotations, which may vary slightly between studies and may not capture tumors that span both regions or junctional cancers. Additionally, proximal and distal groups might have other compounding differences; for instance, proximally located tumors in our cohorts could have had a higher proportion of advanced stage or different treatment histories than distal tumors. We attempted to mitigate confounding by focusing on transcriptional regulators consistently identified by two independent analytic pipelines, but we cannot infer causality from these correlations alone. Another limitation is that the master regulator analysis (whether via corto or RegEnrich) is a computational approach that infers regulatory influence from expression patterns and network information; thus, the predicted MRs (FOXM1, STAT3, GATA6, CDX2, etc.) should be validated experimentally. We did not functionally test these transcription factors in vitro or in vivo, and it remains to be confirmed that modulating these MRs can reproduce the location-specific phenotypes. Finally, our pathway enrichment findings, while biologically plausible, are broad; pathways like NF-κB signaling or metabolic processes encompass many genes, and further work is needed to pinpoint the exact effectors driving the aggressiveness of PGC or the more indolent nature of some DGC. To address this limitation, future studies could incorporate pathway refinement techniques aimed at enhancing interpretive resolution. Tools such as GSEA pruning, leading-edge analysis, or clustering-based dimensionality reduction (e.g., EnrichmentMap or semantic similarity filtering via the clusterProfiler R package) can help distill broad enrichment results into more functionally cohesive gene modules. These strategies allow for the identification of non-redundant pathways and prioritize core gene subsets most relevant to phenotype stratification. While not employed in the present analysis to maintain focus on master regulator inference, the adoption of such methods in future work could yield more granular insights into the transcriptional programs underpinning the biological divergence between PGC and DGC.

Looking forward, our study opens several avenues for future research. Prospective studies should be conducted to validate these location-specific transcriptional programs in independent patient cohorts, ideally with uniform criteria for tumor location and comprehensive clinical data. It would be valuable to integrate additional data types, for example, epigenetic profiles (to see if promoter methylation of genes like GATA6 or CDX2 differs by location) [24] or proteomic analyses (to confirm activation of STAT3, NF-κB, etc., at the protein level in PGC) [62]. Mechanistic studies using organoid or cell line models from proximal and distal tumors could clarify the functional roles of key regulators. For example, knocking down FOXM1 or STAT3 in proximal models may reduce tumor growth [30], while overexpressing CDX2 or GATA6 could induce a more differentiated, less aggressive phenotype, advancing therapeutic understanding and precision medicine [63]. Such hypotheses could be tested in gastric cancer organoids or patient-derived xenografts, which preserve key histological and molecular features and allow functional assessment of transcriptional reprogramming in a controlled or in vivo setting. Another intriguing direction is to investigate the upstream drivers of the differential MR activity. Our data and prior literature suggest that the tumor microenvironment might play a role, e.g., cytokine signaling (IL-6/STAT3) might be more pronounced in obesity-associated cardia cancers, whereas chronic H. pylori infection and associated epigenetic changes (like GATA2 silencing) could facilitate the GATA6/CDX2 program in distal cancers [14,52]. Our findings support using location-specific molecular markers for risk models and stratified clinical trials. Proximal and distal gastric cancers differ biologically, not just anatomically, highlighting the need for personalized treatment approaches and improved prognostication based on tumor location and underlying molecular features.

## 5. Conclusions

This study highlights the importance of tumor location in gastric cancer biology. Transcriptomic analyses revealed distinct master regulators for proximal and distal tumors. Proximal tumors showed increased FOXM1 and STAT3, linked to poor survival, while distal tumors expressed differentiation markers like CDX2 and GATA6, associated with better outcomes. These findings not only highlight novel biomarkers and therapeutic targets but also suggest that integrating tumor location and master regulator profiles could refine clinical decision-making, guide risk stratification, and inform the design of future personalized clinical trials in gastric cancer.

## Data Availability

The datasets analyzed in this study are publicly available through the following sources: The Cancer Genome Atlas (TCGA): https://portal.gdc.cancer.gov (accessed on 15 February 2025); Gene Expression Omnibus (GEO): GSE62254 and GSE15459, accessible at https://www.ncbi.nlm.nih.gov/geo (accessed on 1 March 2025).
